# Editorial Note: Viral Communities Associated with Human Pericardial Fluids in Idiopathic Pericarditis

**DOI:** 10.1371/journal.pone.0348724

**Published:** 2026-05-06

**Authors:** The PLOS One Editors

The *PLOS One* Editors issue this notice to update the previously published Expression of Concern on this article [1,2].

Following the publication of the article and Expression of Concern [1,2], PLOS investigated concerns pertaining to the reported ethical approval and the article’s adherence to *PLOS One*’s research ethics policies.

Specifically, this study reports the use of pericardial fluid samples collected from human participants, including minors, between 2007–2010. The Ethics Statement reports that the study was approved by the local ethics committee IFR48 (Marseille, France) and that written informed consent was obtained from the patients or their legal guardians, but the article does not report an ethics approval reference number. The Expression of Concern [2] was issued due to concerns about several approvals issued by this institution and concerns that the article did not report approval from a Comité de Protection des Personnes (CPP).

A representative of the Aix-Marseille Université Ethics Committee stated that the institutional investigation into the ethics concerns concluded this article meets ethical standards. They commented that the study consists exclusively of a retrospective analysis of pericardial fluid samples collected as part of routine patient care, and that the study did not require ethics approval from a Comité de Protection des Personnes according to French law. The representative provided the ethics approval document #09–011 for editorial review.

Ethics approval document #09–011 was issued by the Comité d#39;Éthique de l#39;Institut Fédératif de Recherche de la Faculté de Médecine de Marseille (IFR48, Marseille, France) on July 21, 2009, for a study titled “*Viral metagenomics of human pathologies with unknown ethiology*”. It allows for the use of cerebrospinal fluids, pericardial fluids, pulmonary biopsies, and blood stored at the bacteriology laboratory, and the study described in the document appears to match the study reported in this article.

However, in investigating this matter PLOS identified potential competing interests between the IFR48 ethics committee that granted the ethics approval and one or more of the article#39;s authors, including concerns that one of the authors of this article is listed as a member of the ethics board who issued the ethics approval. In light of this finding, PLOS is unable to confirm that this study was reviewed by an independent ethics board. Furthermore, PLOS has not been able to secure input from an independent official confirming whether or not this study should have been subjected to CPP approval as per French legislation.

In light of the unresolved issues, the Expression of Concern stands.
